# A Case of Acute Transverse Myelitis in a Mildly Symptomatic Patient: An Emerging and Serious Neurological Manifestation of COVID-19

**DOI:** 10.7759/cureus.24222

**Published:** 2022-04-17

**Authors:** Elvina C Lingas

**Affiliations:** 1 Internal Medicine, Presbyterian Hospital, Albuquerque, USA

**Keywords:** covid-19, sars-cov2, acute myelitis, myelitis, acute transverse myelitis (atm)

## Abstract

Coronavirus disease 2019 (COVID-19) is known to have neurological manifestations and one of them is acute transverse myelitis (ATM). Despite being exceedingly rare (1.34-4.6 cases per million/year), COVID-19-associated ATM cases have continuously been reported and have significant health impact to patients. This case report presents a previously healthy, unvaccinated male who developed COVID-19-associated ATM.

## Introduction

The coronavirus disease 2019 (COVID-19) due to severe acute respiratory syndrome coronavirus 2 (SARS-CoV-2) since its first emergence in Wuhan city, China, in December 2019, has continuously become a major issue in global pandemic. More variants have emerged with increasing virulence. Cases have reached over 300 millions with around 5 millions deaths. COVID-19 is known to have neurological manifestations such as acute cerebrovascular disease, impaired consciousness, and skeletal muscle injury. In a retrospective study performed at Wuhan in 2019, it was seen that more neurological manifestations were found in groups with severe infection [1].

There is no experimental data so far that is able to elucidate the exact mechanism of COVID-19 in the nervous system. It is important to note that COVID-19 is not a neurotropic virus; although it does bind to angiotensin-converting enzyme 2 (ACE2) receptors, which can also be found in glial cells and neuron, and further initiates viral replication and immune mediated direct damage to neurons [2].

Transverse myelitis (TM) is characterized by spinal cord dysfunction with multitudes of symptoms such as paresis and sensory level and/or autonomic (bladder, bowel, and sexual) impairment below the level of the lesion. Prognosis remains variable with the cause of the transverse myelitis carries a significant impact. As such, post-vaccination myelitis, for example, carries a very different prognosis than myelitis brought on by chronic illness [3].

## Case presentation

A 70-year-old previously healthy, unvaccinated male presented with two days history of numbness on both of his legs from the waist down that made it difficult for him to walk. He did not have any fever or chills; however, he did complain of mild headaches. Symptoms initially started on his feet that progressed upwards. He had flu-like illness two weeks before the symptoms started and he tested positive for COVID-19 via polymerase chain reaction (PCR) test during that presentation; however, he reported that he had not sought medical treatment then. Physical exam showed hyperreflexia and symmetrical motor and sensory deficits. He presented to a community hospital initially and in the ED he was found to have urinary retention and a foley catheter was placed. Urinalysis did not show any sign of infection. He was found to have normal vital signs, laboratory workup showed normal WBC and intact liver and kidney function. MRI of cervical, thoracic, and lumbar spine did not show any suspicious enhancements. MRI brain did not show any abnormality as well. Lumbar puncture was performed and CSF analysis showed 453 nucleated cells with 75% neutrophils, 10 RBC, glucose of 51, and protein of 122.8. CSF gram stain and final culture were both negative. He was treated empirically with intravenous ceftriaxone, ampicillin, acyclovir, high dose methylprednisolone, and immunoglobulin. Due to the lack of available inpatient neurology services, he was transferred to our tertiary care hospital for further care.

He was seen by Neurology in our tertiary care hospital and CSF was repeated. Due to pleocytosis in the previous CSF analysis and a history of headache, Infectious Disease (ID) was also consulted due to concern of bacterial encephalitis and recommended to continue empiric antibiotics while waiting for the repeat CSF analysis, The repeat CSF showed elevated protein of 93, glucose 68, elevated cell count of 93, neutrophils 8, lymphocytes 75, monocytes 17. A repeat MRI of the entire axis was done and again no enhancement was seen. The patient was started on prednisone taper by Neurology. Rapid plasma reagin (RPR), CSF venereal disease research laboratory slide test (VDRL), coccidioides antibody panel, fungal antibody panel, and cryptococcal antigen were all negative. Enterovirus and varicella zoster PCR was negative. Neuromyelitis optical (NMO) antibody and paraneoplastic antibody were negative as well. His West Nile antibody was equivocal; however, as per the patient, he had a West Nile infection years ago. The patient was maintained on intravenous ampicillin as per ID recommendations, which was discontinued after the Listeria antibody was found to be negative.

The patient had some improvement in his paresis while in hospital and was discharged to acute inpatient rehab to facilitate his needs for physical therapy. During his inpatient stay, he remained asymptomatic in room air, and he did not receive any treatment for COVID-19.

## Discussion

Various neurological complications have been reported with COVID-19 and they range from dizziness and headaches to stroke, Guillain Barre syndrome, and myelitis [4-6]. There are many possible pathophysiological pathways that could be responsible for this. It has been well documented that COVID-19 directly invades the CNS system [7]. COVID-19 can recognize and bind ACE2 receptors in our CNS system. In a 2020 study, it was shown that the COVID-19 virus has a 10-20 fold increase in the expression of ACE2 binding protein compared to other coronavirus variants [8]. Severe pneumonia-causing hypoxia can also cause indirect damage to the CNS system [9]. The virus can also induce cytokine storms, which increase levels of inflammatory cytokines and activation of T lymphocytes, macrophages, and endothelial cells. Furthermore, releases of interleukins 6 cause vascular leakage, activation of complement and coagulation cascade, disseminated intravascular coagulation, and end-organ damage including stroke [10,11].

In a case series by Mao et al., it was shown that severely ill groups had a higher incidence of neurological complications (40 (45.5%) Vs. 38 (30.2%)). The CNS complications ranged from dizziness to stroke, although no specific myelitis complication was reported [1].

In a 2021 case report and clinical review of 43 ATM patients, Gustavo et al. reported that cases of ATM had a latency of 10 days to six weeks, which they postulated as possible post-infectious neurological complications mediated by the host’s immune system. This is similar to our patient’s presentation. The mean age of that review was reported to be 49 years old. All patients had typical features of ATM with acute onset of paralysis, sensory level, and sphincter deficits [12].

According to the Transverse Myelitis Consortium Working Group, to diagnose TM, an appropriate clinical picture of spinal cord symptoms should be confirmed with contrast enhancement of MRI of the spinal cord and/or elevated protein on the CSF. MRI should be performed to rule out compression of the spinal cord as well as an MRI brain to rule out any brain lesion [13]. There has not been a case report of COVID-19-related myelitis with normal MRI spine as documented in our patient. It is unclear if the lack of enhancement on the MRI spine has any correlation to the favorable outcome. In a systematic review of 21 COVID-19 cases with spinal cord complications, most of the patients improved and the mortality rate was less than 10% [14].

High-dose intravenous glucocorticoids remain the standard of care in patients with TM. Treatment should be given as soon as possible when there is strong clinical suspicion. Preferred regimens are methylprednisolone (30 mg/kg up to 1000 mg daily) or dexamethasone (120 to 200 mg daily for adults) for three to five days [15]. Plasma exchange may be indicated for cases that do not respond to steroids. In a Mayo clinic randomized trial of patients with acute, severe neurological deficits caused by multiple sclerosis or other inflammatory demyelinating diseases of the CNS who fail to recover after treatment with high-dose corticosteroids, plasma exchange led to at least moderate functional recovery [16]. There is a role of immunosuppressive therapy in cases of myelitis caused by systemic autoimmune diseases such as systemic lupus erythematosus or multiple sclerosis but it is tailored to the primary cause.

## Conclusions

ATM in COVID-19 is not an uncommon neurological complication. It is still unclear if this is a marker of severe COVID-19 since it has happened in both severe and milder cases. Clinicians should assess any neurological complaint presented by patients with COVID-19 and implement early appropriate workup if necessary.
